# An update on bloodborne viruses among people who inject drugs in South Asia

**DOI:** 10.1186/s12954-026-01433-x

**Published:** 2026-03-10

**Authors:** Md Ferdous Rahman, Md Sharful Islam Khan, Muhammad J. A. Shiddiky, M. Mamun Huda, Utpal K. Mondal, Nusrat Jahan, Shakeel Mahmood, Allen G. Ross

**Affiliations:** 1 Rural Health Research Institute, Charles Sturt University, Orange, NSW Australia; 2 Programme for HIV and AIDS, Health Systems and Population Studies Division, International Centre for Diarrhoeal Diseases Research, Dhaka, Bangladesh; 3 School of Rural Medicine, Charles Sturt University, Orange, NSW Australia; 4 Department of Public Health, North South University, Dhaka, Bangladesh; 5 College of Medicine, Ajman University, Ajman, United Arab Emirates

**Keywords:** Bloodborne viruses (BBVs), People Who Inject Drugs (PWID), HIV, Hepatitis B (HBV), Hepatitis C (HCV), South Asia, Disease Burden, Harm Reduction

## Abstract

Situated between two of the world’s largest opium-producing regions, South Asia faces significant public health threats associated with drug trafficking and injecting drug use. People who inject drugs (PWID) in South Asia experience disproportionately high rates of bloodborne viruses (BBVs), including human immunodeficiency virus (HIV), hepatitis B virus (HBV), and hepatitis C virus (HCV). These high rates are driven by factors such as drug trafficking routes, socioeconomic marginalization, poor surveillance, and inadequate harm reduction services. Pakistan has the highest reported prevalence rates, with HIV and HCV rates exceeding 30% and 50%, respectively, while India, Bangladesh, and Afghanistan report localized epidemics in urban and border areas. Co-infections, particularly HIV/HCV, further complicate clinical management and public health responses. Despite the implementation of needle-syringe programs and opioid substitution therapy in several countries, service coverage remains below recommended levels due to legal, financial, and structural barriers. Marginalized subgroups, including women and incarcerated individuals, remain underserved and often overlooked. In this review, we discuss the burden of these infections among PWID in South Asia, current control strategies, and the precarious future given the recent instability to the USAID, PEPFAR, WHO, and the Global Fund by the Trump administration.

## Introduction

Injecting drug use is a significant global health challenge, afflicting approximately 14·8 million people aged 15–64 years worldwide, including an estimated 2·8 million women, and 0·4% transgender [1]. The sharing of contaminated needles is a major driver of bloodborne viruses (BBVs), including human immunodeficiency virus (HIV), hepatitis B virus (HBV), and hepatitis C virus (HCV) transmission among people who inject drugs (PWID). Global estimates from 1990 to 2016 indicate high prevalence rates of HIV (17.8%), HCV (52.3%), and HBV (9.0%) among this group [2]. Beyond mono-infections, multi-morbidity is common; later studies (2008–2018) suggest that 13% of PWID experience HIV/HCV co-infection, while roughly 2% face HIV/HBV or triple co-infections involving all three viruses [3].

South Asia, encompassing Afghanistan, Bangladesh, Bhutan, India, Maldives, Nepal, Pakistan, and Sri Lanka, is home to nearly a quarter of the world’s population and is uniquely positioned between two prominent opium-producing zones, the 'Golden Crescent' to the west and the 'Golden Triangle' to the east. This geographic proximity has entrenched the region within global drug trafficking networks, turning several South Asian countries into both transit corridors and growing consumption zones for injectable illicit substances. These dynamics have accelerated the spread of injecting drug use and contributed to a rising burden of BBVs, among PWID in the region [4, 5].

While country-level studies and surveillance reports have generated valuable data, the evidence base in South Asia remains fragmented across settings, methodologies, and programme sources, limiting coherent regional interpretation and synthesis. To address this gap, we conducted this narrative review of epidemiological, surveillance, and programmatic evidence on HIV, HBV, and HCV among PWID in South Asia. An electronic search was undertaken using terms such as “PWID,” “HIV,” “hepatitis C,” “hepatitis B,” “harm reduction,” and the names of individual South Asian countries across PubMed, Scopus, Web of Science, and Google Scholar, supplemented by targeted retrieval of non-peer-reviewed but data-bearing grey literature, including Integrated Biological and Behavioural Surveillance (IBBS) reports, national HIV programme documents, and technical reports from WHO, UNAIDS, UNODC, and Harm Reduction International. Searches covered the literature available up to 31 December 2025, without restriction on the starting year. Evidence was synthesised across predefined analytic domains encompassing BBV burden, co-infection patterns, and harm-reduction coverage and policy responses, including shifts in international financing. Where available, prevalence estimates were prioritised from national surveillance systems; when unavailable, we used the most robust PWID-specific estimates from global systematic reviews. Given heterogeneity in study design, time period, and surveillance context, results are presented descriptively rather than statistically pooled, and interpretation considers key limitations of PWID evidence, including surveillance and site selection bias, non-response and recruitment-related bias, and variable reporting quality in grey literature; older estimates are included to characterise historical trajectories and should not be interpreted as current burden in the absence of recent, methodologically comparable surveillance.

## Burden of injecting drug use in South Asia

### Prevalence and population estimate of injecting drug use

Injecting drug use presents a growing public health challenge in South Asia, with prevalence rates substantially exceeding the global average of 0.33% among adults aged 15–64 years [1]. A regional estimate reported in 2020 suggests that intravenous drug use may affect approximately 2.5% of individuals in this region, reflecting both the scale of illicit drug markets and underlying socioeconomic vulnerability [6].

**Fig. 1:**
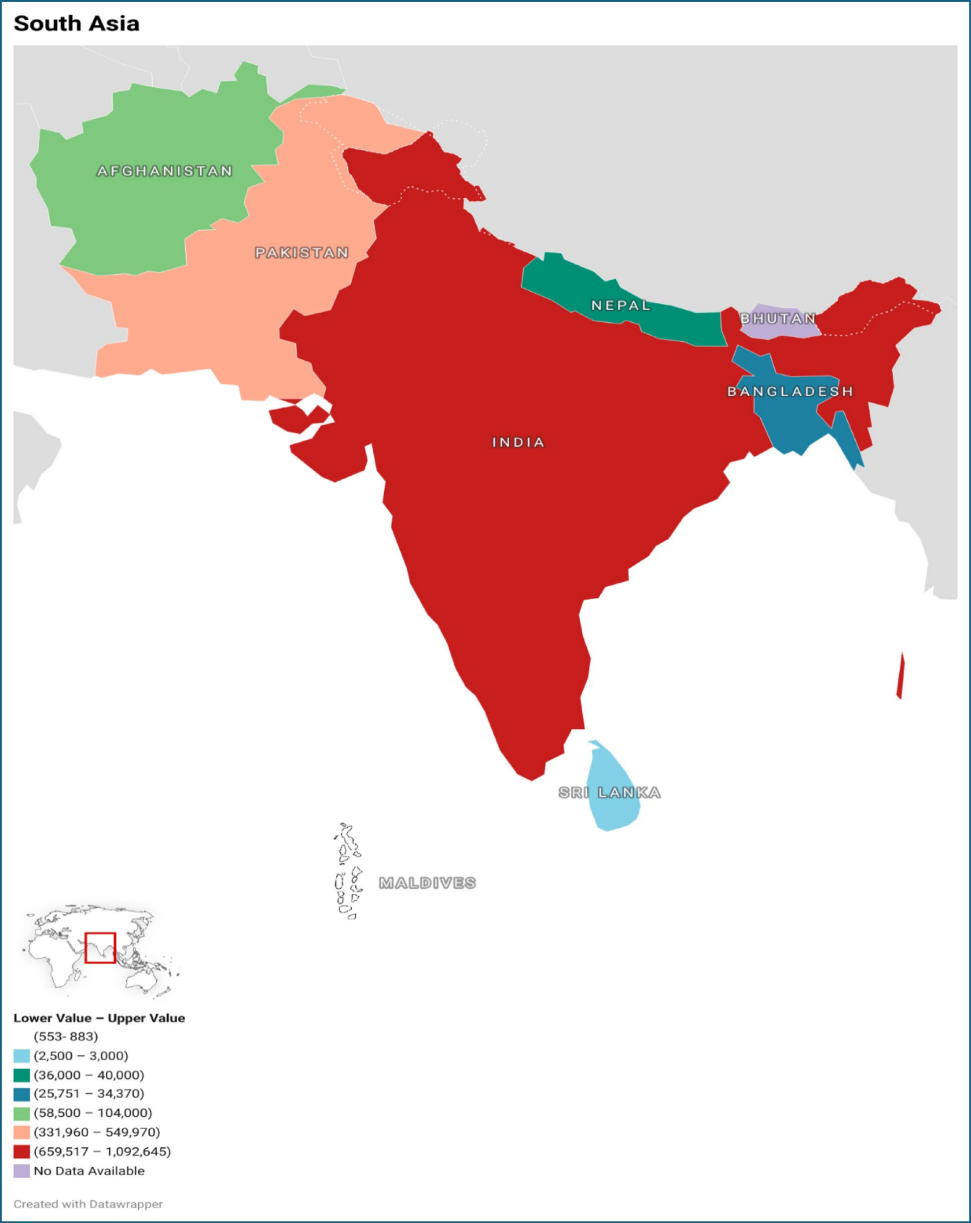
Country-wise estimates of people who inject drugs in South Asia

Estimates of PWID vary across South Asian countries due to differing surveillance capabilities, data collection methodologies, legal frameworks, and sociopolitical conditions. Findings from a 2025 multi-stage population size estimation analysis indicate that India hosts the largest population of PWID in South Asia, estimated at approximately 850,000 (range: 659,517–1,092,645). This is followed by Pakistan with 430,000 (range: 331,960–549,970) and Afghanistan with approximately 80,000 PWID (range: 58,500–104,000) (Fig. 1). Smaller yet substantial PWID populations have been documented in Nepal (38,000, range: 36,000–40,000) and Bangladesh (30,000, range: 25,751–34,370), whereas markedly lower estimates have been reported for Sri Lanka (2700, range: 2500–3000) and Maldives (700, range: 553–883) [7]. Bhutan, however, was not included in these regional estimates, and no formal population size data are currently available.

### Demographic and socioeconomic characteristics

The demographic profile of PWID populations in South Asia predominantly consists of young adults, primarily males between 25 and 35 years. Data indicate that initiation into injecting drug use commonly occurs during adolescence or early adulthood, mirroring global trends and underscoring vulnerabilities associated with youth, including peer pressure, unemployment, and inadequate awareness about harm reduction [1, 8]. While the majority of PWID in South Asia are male, women and adolescents represent a growing but often overlooked subgroup.

Women who inject drugs (WWID) constitute approximately 3.1% of the regional PWID population, a figure significantly lower than estimates from North America (30.4%) and Australasia (34.8%) [1]. Although this may partly reflect lower prevalence, it is also likely influenced by underreporting due to stigma, discrimination, and lack of gender-sensitive surveillance and outreach [9].

Socioeconomic disadvantage is a defining feature of PWID communities in this region. Across India, Pakistan, Bangladesh, and Nepal, high unemployment, low educational attainment, and widespread illiteracy are common, particularly in urban and peri-urban settings. Many rely on informal livelihoods such as daily wage labour, waste collection, or street vending. Housing insecurity is also prevalent, with many living in slums or public spaces without basic sanitation or health services. These conditions are closely linked to unsafe injecting practices, including needle sharing and the use of non-sterile equipment, and reflect broader barriers to harm reduction and healthcare access [8–10].

### Geographical disparities within countries

PWID populations in South Asia are highly concentrated in urban centres, on international borders, and transit hubs. These areas often intersect with major drug trafficking routes [11–13]. In Pakistan, major cities, including Karachi, Lahore, Faisalabad, and Peshawar, report rising PWID populations, reflecting the influence of local drug trafficking routes, the availability of injectable substances, and socioeconomic marginalization [14]. India’s northeastern states, particularly Manipur, Mizoram, and Punjab, show significantly higher injecting rates due to their proximity to cross-border heroin trafficking routes [4]. In Bangladesh, Dhaka and the surrounding metropolitan areas show concentrated PWID populations linked to urban poverty and economic disparities [15]. Nepal also shows geographic clustering, especially in Kathmandu and the Far Western region, often associated with seasonal labour migration and returnee populations from India [16].

### Injecting drug use patterns and risk behaviours

Injecting drug use in South Asia remains predominantly opioid-based, with approximately 88·7% (range: 82.6–93.4%) of PWID reporting opioids as their primary injected substance. Heroin is the primary opioid injected in Pakistan, Afghanistan, and India, influenced directly by extensive opium producing and trafficking networks from the neighbouring’Golden Crescent’ region, including Afghanistan and Pakistan [5, 17]. In contrast, pharmaceutical opioids, such as buprenorphine and pentazocine, are frequently injected in other parts of India, Bangladesh, and Nepal, often sourced from diverted medical supplies or local illicit pharmaceutical markets [18]. Compared to opioid use, stimulant injection, particularly amphetamine-type stimulants (ATS), is less common, yet nearly one in four PWID (24.1%, range: 12.1–38.6%) report injecting ATS, particularly in urban settings where stimulant markets are expanding [1]. Use of polydrug is reported in the region with common co-administration of opioids alongside benzodiazepines, antihistamines, and alcohol, commonly referred to as the ‘South Asian cocktail’, which heightens overdose risks and injection-related complications [19]. Daily injecting is also highly prevalent, with an estimated 85·2% (95% CI: 81.8–89.0%) of PWID injecting once or more per day. This is among the highest rates globally, exceeded only by Latin America and East and Southeast Asia. These injection risks are compounded by sexual vulnerabilities, with 21.9% reporting unprotected sex with casual partners and 11.9% engaging in sex work. Importantly, these risks are not uniformly distributed across genders. WWID face compounded risks due to both unsafe injecting and unprotected sexual exposure. Engagement in sex work is common among WWID, and this subgroup is more likely to share injecting equipment, have unprotected sex with clients or intimate partners, and report higher rates of sexually transmitted infections compared to male PWID [1, 20].

## Prevalence of bloodborne viruses among PWID in South Asia

### HIV prevalence among PWID in South Asia

While the overall population prevalence of HIV remains low (below 1%) across South Asia, the prevalence among PWID is disproportionately high, with considerable national and sub-national variations [21]. Among countries in the region, Pakistan is the most severely affected, with an HIV prevalence of 38.4% among PWID reported in 2017, the most recent publicly available national surveillance estimate, representing one of the highest documented burdens globally (Table 1). Major urban centres, including Karachi, Lahore, and Faisalabad, report prevalence rates between 20% and 40%, reflecting deeply entrenched structural challenges. The 2019 HIV outbreak in Larkana highlighted the consequences of inadequate needle and syringe programmes (NSP), limited availability of antiretroviral therapy (ART), and weak health system coordination [12].

**Table 1:** Prevalence of HIV, HCV antibody, and HBsAg among people who inject drugs in South Asia

Countr	HIV Prevalence (%)	HIV source (Year)	HCV Ab Prevalence (%)	HCV Source (Year)	HBsAg Prevalence (%)	HBsAg Source ( Year)
Afghanistan	4.4	IBBS (2012) [25]	37.5	Global Syst. Review (2012–16) [1]	5.5	Global Syst. Review (2012–16) [1]
Bangladesh	4.1	IBBS (2020) [24]	33.2	IBBS (2020) [24]	7.0	Global Syst. Review (2013) [1]^†^
Bhutan	No Data	–	No Data	–	No Data	–
India	9.0	National Sentinel Surv. (2021) [23]	33.4	National Sentinel Surv. (2021) [23]	3.0	National Sentinel Surv. (2021) [23]
Maldives	ND		ND		ND	
Nepal	2.8	IBBS (2021) [32]	13.3	IBBS (2021) [32]	0.8	IBBS (2021) [32]
Pakistan	38.4	IBBS (2017) [33]	54.5	Global Syst. Review (2012–17) [1]	7.9	Global Syst. Review (2017) [1]^†^
Sri Lanka	0	IBBS (2018) [34]	6.2	IBBS (2018) [34]	0.1	IBBS (2018) [34]

*HCV Ab* Hepatitis C virus antibody; *HBsAg* Hepatitis B surface antigen; *IBBS* Integrated biological and behavioural surveillance;

Citation numbers in brackets correspond to the reference list. Single-year values represent a single survey round, while year ranges indicate pooled estimates from systematic reviews. Estimates reflect predominantly or exclusively male PWID, as female PWID are frequently underrepresented or excluded from aggregate analysis due to insufficient sample sizes

^†^HBsAg estimates for Bangladesh and Pakistan were derived from the global systematic review [1] but were based on single country-specific primary studies conducted in 2013 and 2017, respectively

In contrast, India presents a complex epidemiological landscape marked by regional heterogeneity. While national HIV prevalence among PWID in India was reported at approximately 9% in 2021, northeastern states such as Manipur and Mizoram have consistently exhibited substantially higher prevalence relative to national levels. Manipur's prevalence peaked at nearly 50% in the 1990s but declined significantly due to harm reduction scale-up. However, Mizoram continues to report a prevalence rate above 20%, highlighting persistent gaps in equitable service delivery and inconsistent regional implementation of national policies [4, 22, 23].

Bangladesh provides another illustrative example of fluctuating trends influenced by the intensity of interventions. In Dhaka (the capital of Bangladesh), HIV prevalence among PWID rose sharply to 22% in 2016 before falling to 4.1% by 2020, largely due to targeted harm reduction programs. However, the most recent surveillance (IBBS 2020) indicates that certain districts continue to record prevalence rates above the national average. In Dhaka, the estimate stands at 6.4%, while Narayanganj, an adjacent district to the capital, reports 6.7%, with both figures exceeding the national mean [24]. These figures signal ongoing transmission, further underscored by 2023 data showing that PWID account for 13% of all new HIV infections nationally [13].

Data availability for Afghanistan remains limited, with the last national surveillance in 2012 reporting a prevalence of 4.4% among PWID [25]. Sub-national surveys conducted in 2019–2020 reveal significantly higher rates in key urban centers based on self-reported data; for example, HIV positivity was 0% in Zarang, 27.1% in Jalalabad, and as high as 63.2% in Kabul [26]. These disparities are largely attributed to ongoing conflict, a weakened healthcare infrastructure, and limited access to harm reduction services, especially NSP and Opioid Substitute Treatment (OST) or Opioid Agonist Therapy (OAT) [8, 27].

Nepal has shown encouraging trends, with HIV prevalence among PWID in Kathmandu declining from over 50% in the early 2000s to 2.8% in recent years (2021), largely due to community-led harm reduction initiatives. These approaches extend beyond biomedical services to incorporate holistic, community home-based care models that address mental health, substance use, and ART adherence. Evidence shows that such interventions significantly reduce depressive symptoms, anxiety, stress, and substance use among HIV-positive individuals, while improving adherence to ART through structured home visits, peer support, family counselling, and psychosocial engagement [28]. However, gender disparities persist; studies show a higher prevalence among female PWID (8.8%) [29, 30].

The Maldives, Sri Lanka, and Bhutan have not reported any injection-related outbreaks [31].

### Viral hepatitis burden among PWID in South Asia

PWID in South Asia carry a substantial burden of viral hepatitis, particularly HCV and HBV. Both infections are associated with severe long-term health outcomes, including cirrhosis and hepatocellular carcinoma, especially in the absence of timely diagnosis, antiviral treatment, and harm reduction services are lacking [35]. HCV prevalence among PWID in South Asia is significantly higher than in the general population (0.3–1.5%), representing one of the most persistent threats to this group’s health [7]. As with HIV, Pakistan bears the highest rates of HCV among PWID in the region, with a national prevalence estimated at 54.5%. This prevalence varies markedly across urban settings, peaking as high as 94.3% in densely interconnected injecting networks in cities such as Karachi [7, 36].

India follows closely, with a national HCV prevalence estimated at 33.4% in 2021 [23]. However, this national figure masks extreme regional variations. In northeastern districts such as Churachandpur (Manipur), HCV prevalence has historically exceeded 90% **(2009–2010)**, reflecting long-standing patterns of opioid injection and deficiencies in harm reduction services [37]. More recent surveillance underscores that high-burden pockets persist beyond the northeast; for instance, HCV prevalence among PWID in Uttar Pradesh reached 73% in 2021, reflecting continued gaps in NSP coverage, limited integration of hepatitis testing within HIV programmes, and poor treatment uptake [38].

Similarly, Bangladesh exhibits sub-national disparities in HCV prevalence among PWID. Although national estimates have remained at approximately **33%**, localized epidemics are markedly more severe. In Chapai Nawabganj, a northwestern border district, HCV prevalence reached 97.5% in 2011, before declining to 68.5% in the most recent IBBS conducted in 2020. In contrast, prevalenc**e** In Dhaka, prevalence remained comparatively stable (39.6% in 2011; 32.6% in 2020), despite consistent harm reduction coverage. These findings highlight the need for region-specific control strategies [7, 15, 24].

In Afghanistan, HCV prevalence among PWID is estimated at 37.5%, reflecting widespread heroin use, limited harm reduction infrastructure, and weak surveillance systems. Most injecting occurs in urban centres such as Kabul and Herat, where NSP coverage is minimal, and OST/OAT is largely unavailable. Ongoing conflict and the criminalization of drug use further restrict access to sterile injecting equipment and HCV testing. Routine screening and access to direct-acting antivirals (DAAs) remain limited, with no national hepatitis strategy targeting key populations [9, 27, 39].

Encouragingly, Nepal has shown signs of improvement, with national HCV prevalence among PWID. Decreasing from over 50% to 13.3%, largely due to long-standing harm reduction initiatives [40]. In contrast, Sri Lanka (6.2%) reports low HCV prevalence. Data from Maldives and Bhutan remain unavailable [7, 8].

HBV prevalence among PWID in South Asia is generally lower than that of HCV, but remains a significant concern due to the risk of chronic liver disease and hepatocellular carcinoma [35]. Despite its clinical relevance, HBV remains underreported among PWID in South Asia, primarily due to surveillance limitations. Unlike HIV and HCV, HBsAg testing is often not included in IBBS surveys (as seen in countries like Bangladesh & Pakistan), resulting in critical data gaps [24]. Furthermore, prevalence figures cited in UNODC reports are often derived from older, sub national studies, and only a few countries maintain routine HBV surveillance in key populations [41].

Available data show considerable variation across the region. As summarised in Table 1, reported HBsAg prevalence among PWID varies, ranging from 0.1% in Sri Lanka (IBBS 2018) to 7.9% in Pakistan, based on pooled estimates from studies conducted between 2012 and 2017.

This disparity may be attributed to differences in adult vaccination coverage, harm reduction reach, and injecting practices. Nepal’s community-based interventions and centralized PWID population may offer better programmatic coverage, whereas Pakistan faces systemic implementation barriers [42].

### HIV, HCV, and HBV co-infections

Co-infections among PWID, particularly HIV/HCV and HIV/HBV, pose serious clinical and public health challenges, accelerating disease progression and complicating treatment [43]. Globally, an estimated 82.4% of HIV-positive PWID are co-infected with HCV [44]. However, studies focusing specifically on PWID populations remain limited, often embedded within broader key population surveillance rather than as standalone investigations.

Pakistan reports the highest levels, with studies conducted in Punjab during 2020–2021 documenting HIV/HCV co-infection in over 80% of HIV-positive PWID, reflecting intense overlap of epidemics within dense injecting networks [45]. Following them, Bangladesh reports a 60.7% HIV/HCV co-infection rate among HIV-positive PWID in Dhaka, attributed to inconsistencies in harm reduction coverage and delayed access to ART [43]. India, while reporting a national HIV/HCV co-infection rate of approximately 13% among PWID [46]. Though historically, the highest co-infection rates were recorded in Manipur, with HIV and HCV prevalence among PWID reaching 74.7% and 97.5%, respectively, in 1996. In contrast, lower rates have been observed in western and southern states, though reliable data remain limited. However, evidence on HIV/HCV/HBV triple infections is sparse [38].

In Nepal, available evidence shows stark regional variation. A study in the eastern Terai documented HIV/HCV co-infection rates of up to 65% among HIV-positive PWID, whereas research in Kathmandu Valley reported just 5.6% among female PWID. These discrepancies likely reflect both the localized nature of injecting drug use epidemics and the absence of systematic, nationwide co-infection surveillance [29, 47].

Although evidence on triple co-infection (HIV/HCV/HBV) is limited across the region, global meta-analyses estimate that 2–3% of HIV-positive PWID are triply infected, which poses complex diagnostic and therapeutic challenges [44].

The epidemiological evidence highlights the interconnected burden of HIV, HCV, and HBV among PWID in South Asia. These epidemics are linked to unsafe injecting practices within evolving drug markets dominated by opioids but increasingly involving ATS injection and opioid-ATS polydrug use, while structural factors such as poverty, unemployment, stigma, and criminalisation further exacerbate vulnerability and constrain access to prevention and care, consistent with a syndemic framework [48, 49]. Addressing these overlapping challenges requires comprehensive harm reduction strategies tailored to each country's unique context. The following section examines South Asian responses through harm reduction policies and interventions.

## Response to the burden: harm reduction in South Asia

### Current harm reduction landscape in South Asia

Effective harm reduction interventions, particularly NSP and OST/OAT, are central to preventing HIV, HCV, and HBV transmission PWID. Across South Asia, countries have adopted harm reduction to varying degrees, often within the framework of national HIV prevention strategies [9].

India operates one of the most extensive harm reduction programmes in the region, delivered primarily through the National AIDS Control Programme (NACP). As of the latest data, the network includes over 390 OST/OAT centres and more than 1500 targeted intervention sites, with NSP and OST/OAT forming the core services [38, 50].

Pakistan implements harm reduction primarily through non-governmental and donor-supported initiatives. Needle and syringe programmes operate in 45 district-level Continuum of Prevention and Care (CoPC +) sites and provide outreach in a total of 62 districts. The government offers free hepatitis testing and treatment for people who use drugs and for those unable to afford care, with home delivery of medication to facilitate access [9].

Bangladesh, being one of the earliest adopters of harm reduction in South Asia, with NSPs introduced in 1998 and OST/OAT in 2010 with the financial support from the World Bank, UNICEF, UNAIDS, and the Global Fund, HIV prevention services have expanded significantly through non-governmental organisation partnerships under the leadership of the National AIDS/STD Programme (NASP). The country currently operates 18 OST/OAT clinics, primarily in Dhaka and other urban centres, serving around 2700 clients. NSP services are implemented by non-governmental organisations as part of community-based outreach programmes [15, 24].

Nepal operates harm reduction services through a mix of government and partner-supported initiatives. NSP operates nationwide, delivering an average of 94 sterile syringes per PWID per year, with safe injection reported at over 96%. OST/OAT is delivered at 12 sites across 10 districts, yielding a national coverage rate of approximately 4.3%. These services are supported by the government and international partners such as the Global Fund and ViiV Healthcare UK. The national strategy for viral hepatitis B and C (2023–2030) integrates harm reduction interventions, encouraging collaboration between HIV, hepatitis, and outreach services to enhance prevention and care [51].

Sri Lanka, Bhutan, and the Maldives currently lack formal harm reduction frameworks, reflecting historically low injecting drug use prevalence and punitive drug laws. However, emerging signs of injection drug use, particularly in prisons and urban centres, underscore the need for proactive harm reduction preparedness and surveillance [9].

### Innovations in harm reduction

Amid persistent barriers, several countries have introduced innovative harm reduction approaches tailored to local contexts. India has deployed mobile OST/OAT vans in underserved northeastern states, improving treatment access in remote areas [52], Bangladesh, early integration of digital technology, including telemedicine, has enhanced the accessibility of harm reduction services in settings with limited healthcare infrastructure. Evaluation of telehealth interventions over the 12 months following the shift to telehealth in April 2020 after the national lockdown demonstrated marked improvements in treatment retention (from 14·4% to 87%), reductions in loss to follow-up (from 20 to 8%), and elimination of overdose-related deaths (from 1·3% to 0%) between the pre- and post-intervention periods [53].

### Persistent challenges to effective harm reduction in South Asia

1. *Implementation fidelity and service gaps*
  Despite the formal integration of harm reduction into national HIV prevention frameworks, South Asian countries deliver far below international coverage benchmarks. WHO recommends at least 200 sterile needles per PWID annually, yet the regional mean is 29.4 (95% CI: 26.3–33.4), OST/OAT access is similarly inadequate; although aggregated figures suggest 44.7 out of 100 PWID in South Asia receive OST/ OAT, programme monitoring indicates that only 23% of active PWID were enrolled in OST/OAT in 2022 [9, 54].
  India shows wide subnational disparities. NSP coverage exceeds 90% in some states but falls below 40% in others, with OST/OAT largely urban-centred and minimal in correctional facilities [50]. Nepal’s overall HIV prevention coverage is 67·8%, but OST/OAT uptake is 4·3% and naloxone is restricted to OST/OAT sites despite 94% NSP reach [51]. Bangladesh reaches 59·3% of its UN 2025 target for PWID engagement, with OST/OAT covering only 7·9% of the estimated PWID population. There are no prison-based OST/OAT or NSP services, and naloxone is not available for overdose prevention [55]. Pakistan has no operational OST/OAT, limited NSP coverage, and no formal overdose prevention measures in place. Across the region, prison-based harm reduction is virtually absent, with only a single OST/OAT pilot in one Indian prison and no NSPs in any correctional setting.
2. *Punitive drug laws, crackdowns, and human rights barriers*
  All South Asian countries criminalize drug possession for personal use, with penalties ranging from compulsory detention to long-term imprisonment and even the death penalty for certain trafficking offenses. These legal frameworks contribute to enforcement practices such as police harassment, confiscation of injecting equipment, and targeted arrests near harm reduction sites, which discourage PWID from accessing health services [56, 57].
  The behavioural impact of these policies is significant: approximately 18% of PWID in Bangladesh and 26% in India avoided healthcare in the past year due to stigma and discrimination. Crackdowns in high-prevalence states, such as Manipur and Punjab, have led to declines in NSP attendance and increased unsafe injecting behaviours. Pakistan reports similar patterns, with police harassment documented in multiple provinces [58]. Bangladesh’s anti-drug drives under the Narcotics Control Act 2018 have similarly contributed to healthcare avoidance [59].
3. *Stigma, gender inequities, and exclusion of key populations*
  In addition to legal barriers, pervasive stigma surrounding drug use continues to frame addiction as a moral failing rather than a health issue. WWID face intersecting discrimination based on gender, drug use, sex work, and often HIV status, which leads to lower service uptake despite documented higher HIV prevalence in certain settings [60]. Gender-specific harm reduction services are rare, and prison-based services for women are non-existent. Reliance on male partners for procuring and administering drugs further constrains women’s ability to negotiate safer injecting practices, heightening vulnerability to both parenteral and sexual transmission of HIV and other bloodborne infections [20]. Despite the clear epidemiological rationale, gender-responsive harm reduction programming remains critically underdeveloped across South Asia, leaving the unique needs and risk profiles of WWID largely unmet and perpetuating inequities in health outcomes.
4. *Donor dependence and sustainability risks*
  Harm reduction in South Asia remains chronically underfunded, with most programmes sustained through international assistance, particularly from the Global Fund rather than national health budgets. This over-reliance creates structural fragility: when donor priorities shift or funding cycles end, service coverage contracts sharply, jeopardising continuity of care for PWID. Domestic allocations remain negligible, reflecting persistent political neglect and the failure to embed harm reduction within broader public health financing frameworks [13, 61]. Without sustainable domestic investment, coverage gaps are likely to continue widening, eroding the progress made in recent years. The *2025 UNAIDS World AIDS Day Report* highlights this vulnerability, noting that in countries like Bangladesh and Pakistan, community-led organisations receive 89% of their funding from bilateral partners and less than 0.1% from domestic sources [62]. Without sustainable domestic investment, coverage gaps are likely to continue widening.

## USA funding cuts and implications for the global PWID community

The situation is further exacerbated by US cuts to the US Agency for International Development (USAID), the President’s Emergency Plan for AIDS Relief (PEPFAR), the WHO, and the Global Fund, the SDGs, and the global key population, including PWID, will face a precarious future. The United States has historically provided approximately 32% of total global health financing, making it the largest single donor to international health efforts (Table 2) [63]. Specifically for HIV, in 2023, the United States accounted for 72.6% of total donor government funding for global HIV programs, underscoring its pivotal role in sustaining HIV and harm reduction initiatives. That year, over 90% of international HIV funding originated from just five major donors. By early 2025, all five had announced funding reductions ranging from 8 to 70%, with global HIV financing projected to decline by 4.4% in 2025 and by up to 19.6% in 2026 [64].

**Table 2:** The US global health funding cuts and their impact on key populations

Agency Name	Nature of Funding Reduction	Impact on Key population
USAID	Dissolution of USAID and cancellation of 86% of its global health funding agreements including 71% of HIV-related program award	Termination of harm reduction and HIV prevention services in over 40 low- and middle-income countries, affecting PWID, MSM, sex workers, and migrants. Support for community-led testing, PrEP, and health education was also halted [71]
PEPFAR	Freeze and delay in reauthorization of global HIV programming, with a 90-day freeze on funding and a partial waiver issued later. FY 2026 Budget Request proposed a USD 1.9 billion reduction	Disruptions in ART provision, HIV testing, and harm reduction services; increased risk of ART stockouts; delayed scale-up of PrEP and reduced peer-led outreach and civil society engagement in several settings. The waiver permits only specific activities, including HIV treatment and care, prevention of mother-to-child transmission, and PrEP for pregnant and breastfeeding women [65, 72–75]
WHO	US withdrawal led to a 21 percent cut in WHO’s 2026 -2027 budget, removing about USD 1·1 billion from core funding previously supported by 15 -18 percent US contributions	Major disruption of WHO-supported global programs, including technical support for hepatitis B and C surveillance, laboratory systems, and harm reduction policy development. Budget constraints have led to staff reductions, reduced country-level assistance, and diminished support for BBV responses in low- and middle-income countries [76]
The Global Fund	The US pledged USD 4.6B in November 2025 (vs. the previous USD 6B). A 23% nominal reduction	Potential reduction in OST/OAT, NSP, and community-led HIV services for PWID, particularly in Asia and Eastern Europe; may halt or delay new research, scale-up activities, psychological support, and human rights-based interventions [66, 74, 77]

*PWID* People who inject drugs; *MSM* Men who have sex with men; *ART* Antiretroviral therapy; *PrEP* Pre-exposure prophylaxis; *OST* Opioid substitution therapy; *OAT* Opioid agonis therapy; *NSP* Needle and syringe program; *FY* fiscal year

However, the catastrophic "total withdrawal" scenario feared in early 2025 was partially mitigated later in the year through legislative and multilateral interventions. After the Executive Branch froze aid in early 2025, the US Congress passed a Continuing Resolution in March 2025 that maintained level funding for PEPFAR. In September 2025, the United States released the *America First Global Health Strategy*, which committed to maintaining funding for frontline commodities and covering health worker salaries through early 2026 as bridge support while co-investment increases [65]. In addition, at the Global Fund replenishment conference in November 2025, the United States pledged USD 4.6 billion for the 2026 to 2029 cycle. Although this signalled continued engagement, it represented a reduction from the previous USD 6 billion pledge, indicating partial restoration rather than a return to baseline financing [66].

Despite these mitigation measures, the funding volatility observed in 2025 produced sustained adverse effects on services for key populations, including PWID. According to the WHO, based on estimates from the AIDS Vaccine Advocacy Coalition, approximately 2.5 million individuals who were using pre-exposure prophylaxis (PrEP) in 2024 lost access to it by October 2025 due to disruptions in donor funding [62].

In South Asia, where WHO- and Global Fund-supported systems play central roles in viral hepatitis surveillance and harm reduction delivery, funding volatility has translated into tangible service disruptions that threaten progress toward SDG target 3.3, which aims to end the epidemics of AIDS, tuberculosis, hepatitis, and other communicable diseases by 2030 [67, 68]. External aid remains essential for sustaining HIV prevention and harm reduction infrastructure across the region. In Pakistan, US-funded HIV control activities in Sindh were disrupted, with subcontracted non-governmental organisations losing operational capacity even while hospital-based services continued [69]. For donor-dependent South Asian programmes, reduced replenishment envelopes increase the risk of constrained prevention commodity procurement, given the limited capacity for rapid domestic substitution.

These regional disruptions reflect a broader global pattern. A rapid survey by the International Network of People who Use Drugs found that 25% of 76 community-led organisations worldwide reported losing between 75 and 100% of their harm reduction funding, resulting in the suspension of NSP, OAT, HIV testing, and overdose prevention services [70]. Modelling analyses further suggest that persistent gaps in the international HIV response could contribute to 1.2–3 million additional HIV infections globally between 2025 and 2040, while more severe scenarios involving sustained withdrawal of US-funded programmes project 6.6–15 million additional infections and 4.2–28 million AIDS-related deaths over the same period [64].

## Conclusion

In South Asia, HIV, HCV, and HBV among PWID are not distinct epidemics but part of an entrenched syndemic driven by unsafe injecting and sexual practices, compounded by poverty, stigma, punitive legal frameworks, and underinvestment in harm reduction. Although targeted interventions have achieved some progress, coverage remains uneven, prison-based services are virtually non-existent, and reliance on unpredictable external funding leaves critical programs vulnerable to abrupt contraction. Importantly, women who inject drugs, including those who exchange sex, face heightened risk of bloodborne viral infection while remaining among the least reached by NSP, OAT, and testing and treatment services.

To break this cycle, harm reduction must be embedded within universal health coverage, supported by sustainable domestic financing, and reinforced by legal and policy reforms that remove barriers to care. Services should be gender-responsive, rights-based, with dedicated pathways for women who inject drugs and those who exchange sex and integrated with broader HIV, viral hepatitis, tuberculosis, and mental health strategies. Diversifying funding sources through pooled procurement and strategic partnerships with philanthropic and private sectors is essential for building long-term resilience.

Strengthening the evidence base is equally critical. Surveillance must move beyond irregular cross-sectional snapshots towards repeated, methodologically comparable IBBS rounds at the same sentinel sites, implemented at regular intervals where feasible, using harmonised indicators and comparable methods across cycles. At present, relatively sustained practice is largely limited to India’s national IBBS platform and Nepal’s multi-round PWID IBBS in Kathmandu Valley, whereas many other settings conduct IBBS sporadically, constraining trend inference and programme targeting. Scaling regular, standardised key population surveillance across the region in line with international biobehavioural survey guidance is therefore essential, complemented by longitudinal cohorts, network-based and molecular surveillance, and rigorous evaluation of community-led and digital delivery models in underserved settings. Evaluations of community-led and digital models should focus on fidelity, scalability, and cost-effectiveness in diverse settings, including prisons, rural areas, and among women who inject drugs.

Without decisive political leadership, stable financing, and evidence-informed innovation, South Asia risks perpetuating a preventable cycle of infection, illness, and premature death among an already marginalized population. Such an outcome would undermine progress towards the SDG target of ending AIDS, viral hepatitis, and other communicable diseases by 2030. Given the current evolving funding landscape, this review serves as a timely reference point; future longitudinal monitoring will be essential to quantify the long-term impact of resource contractions on the region’s epidemiological trajectory.

## Data Availability

No datasets were generated or analysed during the current study.

## Abbreviations

AIDS: Acquired Immunodeficiency Syndrome
ART: Antiretroviral Therapy
ATS: Amphetamine-Type Stimulants
BBVs: Bloodborne Viruses
CDIC: Community Drop-In Centre
CoPC +: Continuum of Prevention and Care Plus
DAA: Direct-Acting Antiviral
DIC: Drop-In Centre
HIV: Human Immunodeficiency Virus
HBV: Hepatitis B Virus
HCV: Hepatitis C Virus
HBsAg: Hepatitis B Surface Antigen
IBBS: Integrated Biological and Behavioural Surveillance
LMICs: Low- and Middle-Income Countries
MSM: Men Who Have Sex with Men
NACP: National AIDS Control Programme
NASP: National AIDS/STD Programme
NGO: Non-Governmental Organisation
NSP: Needle and Syringe Programme
OAT: Opioid Agonist Therapy
OST: Opioid Substitution Therapy
PEPFAR: President’s Emergency Plan for AIDS Relief
PrEP: Pre-Exposure Prophylaxis
PWID: People Who Inject Drugs
SDGs: Sustainable Development Goals
UNAIDS: Joint United Nations Programme on HIV/AIDS
UNODC: United Nations Office on Drugs and Crime
USAID: United States Agency for International Development
WHO: World Health Organization
WWID: Women Who Inject Drugs
